# Protocol to establish standards for the elements of infection prevention and control programs and practice and competency standards for infection control professionals in Australian hospitals

**DOI:** 10.1371/journal.pone.0330221

**Published:** 2025-08-14

**Authors:** Ramon Z. Shaban, Deborough Macbeth, Julie Considine, Matthew O’Sullivan, Peter Collignon, Brett G. Mitchell, Donna Waters, Kate Curtis, Phillip L. Russo, Kathy Dempsey, Belinda Henderson, Anne Wells, Sam Butenko, N. Deborah Friedman, Yana Albrey, Christine Gee, Lisa Nicolaou, Frances Sheehan, Mary Wyer, Merrick Powell, Catherine Viengkham

**Affiliations:** 1 Susan Wakil School of Nursing and Midwifery, Faculty of Medicine and Health, The University of Sydney, Camperdown, New South Wales, Australia; 2 Sydney Infectious Diseases Institute, Faculty of Medicine and Health, The University of Sydney, Camperdown, New South Wales, Australia; 3 New South Wales Specialist Service for High Consequence Infectious Disease, Westmead Hospital, Westmead, New South Wales, Australia; 4 Gold Coast Hospital and Health Service, Gold Coast University Hospital, Southport, Queensland, Australia; 5 School of Nursing and Midwifery and Centre for Quality and Patient Safety Research in the Centre for Health Transformation, Deakin University, Geelong, Victoria, Australia; 6 Eastern Health, Box Hill, Victoria, Australia; 7 School of Medicine and Psychology, Australian National University, Canberra, ACT, Australia; 8 Canberra Hospital, Canberra, ACT, Australia; 9 School of Nursing and Health, Avondale University, Cooranbong, New South Wales, Australia; 10 Central Coast Local Health District, Gosford, New South Wales, Australia; 11 School of Nursing and Midwifery, Monash University, Frankston, Victoria, Australia; 12 Emergency Services, Illawarra Shoalhaven Local Health District, Wollongong Hospital, Wollongong New South Wales, Australia; 13 Cabrini Health, Malvern, Victoria, Australia; 14 Clinical Excellence Commission, St Leonards, New South Wales, Australia; 15 Queensland Infection Prevention and Control Unit, Queensland Health, Herston, Queensland, Australia; 16 Department of Health Tasmania, Hobart, Tasmania, Australia; 17 South Australia Health, Adelaide, South Australia, Australia; 18 Victorian Healthcare Associated Infection Surveillance System (VICNISS) Coordinating Centre and Department of Infectious Diseases, University of Melbourne at the Peter Doherty Institute for Infection and Immunity, Melbourne, Victoria, Australia; 19 Northern Territory Department of Health (NT Health), Darwin, Northern Territory, Australia; 20 Toowong Private Hospital, Toowong, Queensland, Australia; 21 Australian Private Hospitals Association (APHA), Barton, ACT, Australia; 22 Infection Prevention Policy Surveillance Unit (IPPSU), Communicable Disease Control Directorate, Department of Health (WA Health), East Perth, Washington, Australia; 23 Canberra Health Services, ACT Government, Canberra, ACT, Australia; 24 NSW Biocontainment Centre, Westmead, New South Wales, Australia; AIIMS: All India Institute of Medical Sciences, INDIA

## Abstract

Healthcare-associated infections (HAI) are the most frequent hospital-acquired complication, resulting in significant mortality, disability, and system-level costs in Australian hospitals. Many HAIs can be prevented with appropriate infection prevention and control (IPC) measures, including IPC programs led by infection control professionals (ICPs). Despite recent improvements in hospital IPC practices in Australia, such as the introduction of National Safety and Quality Health Service (NSQHS) Standards for hospital accreditation, there are currently no evidence-based minimum standards for the content, composition, and governance of IPC programs, nor the identification of their core elements. Furthermore, there is a similar lack of evidence-based minimum standards guiding the practice requirements, skills, and competencies of hospital ICPs. This protocol outlines a sequential three-phase research design to establish the core requirements for the elements and governance of IPC programs, as well as minimum practice standards for ICPs in Australian hospitals. Phase 1 will involve two integrative reviews to synthesise the elements and governance systems of international IPC programs, and the competencies, education and practice standards for hospital ICPs globally. Phase 2 will use survey and interview methodologies to examine the current content and structure of IPC programs and governance systems in Australian hospitals, as well as the academic and professional content of IPC education and training courses for ICPs in Australia. In Phase 3, a modified electronic Delphi study will be conducted to generate expert consensus on the core requirements of Australian hospital IPC programs and systems of governance, and the professional practice, qualifications and competencies for Australian ICPs. The outcomes of Phase 3 will form the basis for the development of new standards that aim to equip the Australian acute care sector to deliver evidence-based IPC practices, education, resources, and governance.

## Introduction

Infections and communicable diseases pose a significant threat to patient safety [1]. Healthcare-associated infections (HAI), such as bloodstream and surgical site infections, are the most frequently occurring complications acquired in hospitals [2,3]. In Australia, 77,402 HAIs (1.4% of hospitalisations) were reported in admitted patients across public and private hospitals in 2022–23 [3]. Recent point prevalence surveys of Australian public hospitals estimate that HAIs contribute to approximately 7,500 deaths and more than 122,500 disability-adjusted life years per annum [4]. HAIs also incur considerable financial and societal costs, increasing the average length of hospital stay by 18.1 days and stretching Australia’s already burdened healthcare system by an estimated $37,000 in extra costs per affected patient in additional and unanticipated care [2]. Moreover, antimicrobial resistance (AMR), epidemics and public health crises, such as pandemics of novel diseases, remain ongoing challenges to the delivery of high-quality and safe healthcare free from HAIs and communicable diseases.

The World Health Organization (WHO) estimates that up to 50% of HAIs can be prevented with appropriate infection prevention and control (IPC) measures [5,6]. Effective IPC is founded upon three core elements: the IPC program [7], the infection control professional (ICP), and the systems of governance that support the program and the professional therein [8,9]. IPC programs are formal, coordinated structures and processes for implementing and evaluating infection control strategies [10]. IPC programs are often comprised of multiple components that operate in tandem to improve IPC practice and reduce HAIs, including formal policies and procedures, risk management, education and training, surveillance, and quality improvement. Central to the IPC program is the appointment of the ICP, a professional who is qualified and responsible for the development, delivery and review of IPC programs. Finally, to support both the IPC program and the professional, there must be formal systems of governance established within the organisation to administer the appropriate authority and scope of both these entities, in addition to evaluating their effectiveness.

Australia has implemented reforms to improve hospital IPC nationally [11,12], including the introduction of National Safety and Quality Health Service (NSQHS) Standards for hospital accreditation [11], national guidelines to improve IPC in acute care settings [12], and professional associations that administer education and credentials for ICPs [13,14]. However, substantial challenges remain due to heterogeneity in how IPC programs are implemented across Australian hospitals, with each State and Territory exercising vastly different oversight and recommendations [7,15–17]. The NSQHS Standards [11], first released in 2011, broadly prescribe actions to guide hospitals in meeting accreditation criteria for IPC. However, these Standards on their own lack sufficient evidence to fully inform the contents and composition of a comprehensive IPC program, including specifications adjusted for hospital profile (e.g., size, location, adult or paediatric, specialist services). The same variability applies to the scope of practice of the ICP. Despite being ubiquitous for most healthcare systems, there are currently no standards that inform the minimum skills, knowledge, and experience required of the ICP, nor are there courses designed to ascribe and confirm their competency [18–20]. Moreover, while initiatives like the Australasian College for Infection Prevention and Control’s (ACIPC) Credentialing Framework provide some structure, credentialling remains non-mandatory and currently less than 100 Australian individuals hold an active credential [13,14].

This research seeks to strengthen hospital IPC practice by establishing minimum standards to inform the governance and composition of hospital IPC programs, as well as the practice requirements and competencies for ICPs. Similar practice standards have been successfully established in emergency nursing [10], which have led to pragmatic recommendations for strengthening the Australian health system including policy, practice, education and research. These standards will ultimately inform IPC materials, hospital IPC programs, education curricula, credentialling requisites, position descriptions and professional performance indicators. Moreover, these recommendations will be evidence-based, consensus-driven by Australia IPC professionals and experts, sensitive to key facility characteristics that may impact practice and, where possible, complement initiatives being completed at national and global levels, such as the WHO’s minimum requirements for IPC programs [21], the current national standards and guidelines [12,22], the existing credentialling framework and educational offerings [14,23].

The aims of this research are to:

1 Establish core requirements for the elements and governance of IPC programs in Australian hospitals (**Stream A**); and
2 Establish minimum practice and competency standards for infection control professionals working in Australian hospitals (**Stream B**).

**Fig 1:**
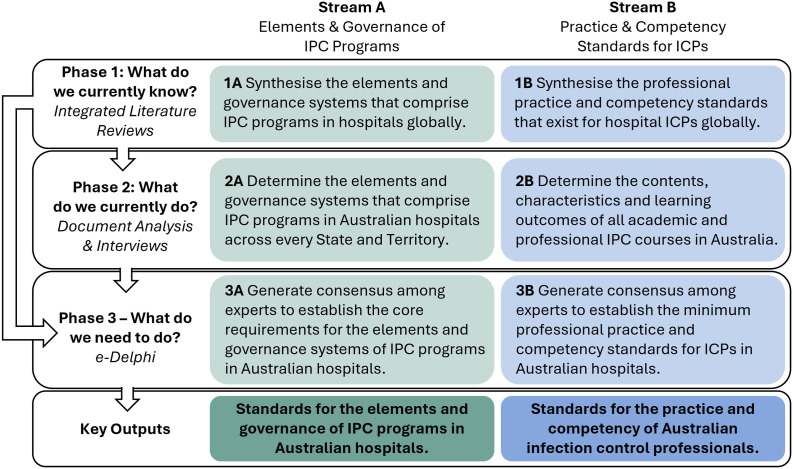
Overview of the three-phase research methodology, which will be completed in parallel across both Stream A and Stream B.

The research aims will be addressed using three sequential study phases (Fig 1). The data collected in each phase will inform the development and analysis of subsequent phases.

- **Phase 1:** Integrative reviews of international literature will be completed to: a) synthesise the elements and governance systems that comprise international hospital IPC programs, and b) synthesise the competencies, education and practice standards for hospital ICPs globally. These reviews will collate existing recommendations and standards for IPC programs and practice, identifying current universal domains of best practice to set the foundations for this study’s outputs and inform both the design and analysis of Phases 2 and 3.
- **Phase 2:** Surveys, document analyses and interviews will facilitate the review of the current contents and structure of: a) IPC programs and governance systems in Australian hospitals, and b) the academic and professional content of IPC education and training courses for ICPs in Australia. In addition to providing a comprehensive snapshot of current IPC systems, Phase 2 will also identify areas for further improvement and reinforcement via interviews with content and practice experts. This knowledge will inform the development of the Delphi items in Phase 3.
- **Phase 3:** A modified electronic Delphi (e-Delphi) process will generate expert consensus for: a) the core requirements for IPC programs and systems of governance in Australian hospitals, and b) the professional practice, qualifications and competencies for Australian ICPs. The outcomes of Phase 3 will form the basis of their respective standards.

## Materials and methods

### Phase 1 – Integrative literature review

#### Objectives.

1. Synthesise the existing elements and governance systems that comprise international IPC programs in hospitals (**1A**).
2. Synthesise the existing professional practice and competency standards and requirements for hospitals ICPs globally (**1B**).

#### Study design.

Two integrative reviews of international literature will be conducted to meet the objectives of each stream. An integrative review method was selected as it is anticipated that secondary sources will comprise a significant proportion of relevant literature. The integrative reviews will be conducted using the methods described by Lubbe et al. (2020) and reported according to the PRISMA-ScR guidelines [24,25].

#### Review search strategy and selection criteria.

Searches will be conducted in multiple databases, including Cumulative Index in Nursing and Allied Health Literature (CINAHL), Scopus, Ovid MEDLINE, Overton and Excerpta Medica Database (Embase). Inclusion criteria will comprise articles written in English and published since 1980. For both streams, secondary sources and grey literature will form vital components of the review. These sources will be identified through a combination of hand searching and citation chaining of relevant reference lists and websites. Websites will be identified and searched using Google and its advanced search function. For each combination of search terms, the top 100 results will be reviewed for their eligible literature. Literature will also be sought from members of the investigation team, which includes numerous members with extensive expertise in IPC practice and programs. Where available, standards for IPC programs and professionals set by global, national organisations or professional bodies (e.g., World Health Organization, Australasian College for Infection Prevention and Control, Association for Professionals in Infection Control and Epidemiology) will also be included. This review will enable the context of the broader issue to be explored in the absence of published literature [26,27]. Articles must describe programs, practices, standards and professional competencies within the hospital setting, and those limited to outpatient clinics and community-based healthcare will be excluded. The broad search terms for both questions are listed in Table 1.

**Table 1:** Search terms for the two integrative literature reviews.

	Review A: Infection Control Programs	Review B: Infection Control Professionals
**Population**	N/A	‘professional’, ‘practitioner’, ‘nurse’, ‘healthcare worker’ ‘long term care’, ‘specialist’, ‘consultant’
**Intervention**	‘program’, ‘standard’, ‘guideline’, ‘polic*’, ‘practice’	‘practice’, ‘standard’, ‘regulation’, ‘guideline’, ‘competenc*’, ‘qualification’, ‘training’,
**Outcome**	‘healthcare associated infection’, ‘infection’, ‘infection prevention’, ‘infection control’, ‘cross infection’	‘healthcare associated infection’, ‘infection’, ‘infection prevention’, ‘infection control’, ‘cross infection’
**Setting**	‘hospitals’, ‘health facilities’, ‘health services’	‘hospitals’, ‘health facilities’, ‘health services’

#### Screening and data extraction.

For each stream, titles and abstracts of identified articles will be independently screened by two reviewers using the online platform *Covidence* [28] (Covidence, Australia, covidence.org). Both reviewers will have expertise in IPC in acute care settings. A pilot screening of a random 10% of articles will be completed to ensure interrater consistency [27]. Two reviewers will then independently complete full-text screening. To minimise reviewer bias, conflicts will be resolved by a third reviewer and/or by group consensus. Data will be extracted and charted by the same two reviewers. A data extraction framework will be adapted from Jones et al. (2015), which has been previously used by the research team [27].

#### Data analysis and synthesis.

A comparative analysis will be conducted in *NVivo*^*TM*^ v14 where common themes and domains for programs and practice standards will be compared across countries [27]. A narrative summary of the results will be constructed, with data being categorised according to recurring themes, and the variations and commonalities between the articles will be summarised [29].

### Phase 2 – Document analysis and key informant interviews

#### Objectives.

1. Determine the current elements and governance systems that comprise IPC programs in Australian hospitals across every State and Territory (**2A**).
2. Determine the contents, characteristics and learning outcomes of all current academic and professional IPC courses available to ICPs in Australia (**2B**).

#### Study design.

- **Stream 2A:** an examination of current IPC programs and governance systems in Australian hospitals will be completed using **surveys and document analysis** to determine the current contents, scope and composition of such programs. This will be followed by semi-structured **interviews** with hospital IPC leads and the creators of existing IPC programs to better comprehend the characteristics of the programs.
- **Stream 2B:** a **document analysis** of available content about IPC education, professional development and training courses will be completed. This will be followed by semi-structured **interviews** with the individuals responsible for the development of those courses. This design has successfully been used to examine the academic and professional characteristics of emergency nursing programs in Australia [30].

#### Stream 2A participant eligibility criteria.

For **Stream 2A**, eligible participants will include all currently or recently employed (within the last 12 months) hospital IPC leads from Australia’s 1,340 public and private hospitals [31]. Individuals with IPC responsibilities but operate under different titles (e.g., quality and safety manager) will also be eligible. To achieve a representative sample, proportionate stratified sampling will recruit participants based on hospital characteristics, including public or private, adult or paediatric, size and geographical remoteness. Stream 2A will aim to obtain IPC programs from at least 30% (n = 403) of all hospitals, to ensure at least 3 hospitals from the Northern Territory (least populous Australian jurisdiction) are surveyed. A convenience sample of 10% (n = 40) of this population will then be recruited for the interviews.

#### Stream 2A data collection and sources.

Data for document analysis of written IPC programs and governance systems will be obtained via an online survey using *REDCap*^TM^ [32,33], a secure web platform for building and managing online databases and surveys managed by the University of Sydney. This survey will be adapted from a version used by Mitchell et al. (2019) [34]. In the survey, participants will answer questions regarding the contents of their organisation’s IPC programs and the relevant governance systems and identify enablers and barriers to effective IPC. Additionally, participants will be invited to provide the latest document version of their IPC programs and governance arrangements. The survey will be open to responses for 10 weeks after the initial invitation is sent. Up to four reminder emails will be sent to participants during this period, with the final reminder to be sent 2 days before the close date. Participants may choose to complete the survey at any time during that period.

Contact details for IPC leads will be obtained from several partner organisations, namely the health departments of each State and Territory for public hospitals, the Australian Private Hospitals Association (APHA) for private hospitals, and the ACIPC for remaining active ICPs. All eligible participants will receive an email invitation containing the participant information package and the REDCap^TM^ survey link. The survey will take approximately 30–45 minutes to complete.

Interview participants will be invited via email, which will contain the relevant participant information package and an interview booking link. Interviews will be conducted on *Microsoft Teams*. The interview will follow a semi-structured guide, developed from previous research [30] and adapted based on Phase 1A findings. Before commencing the interview, the researcher will explain to the participants the interview process, which will include the purpose of the interview, the expected duration, and confirmation that participants approve the recording of the interview. The interview will primarily comprise open-ended questions to explore participants’ perspectives on the current elements of IPC and governance systems, focusing on areas that require further clarification after the Phase 2A Survey. Interviews will be audio recorded, transcribed verbatim and will take approximately 60 minutes to complete. Survey participants will be reimbursed with an AU$30 electronic gift voucher for their participation, and interview participants will be reimbursed with an AU$50 electronic gift voucher.

#### Stream 2A data analysis.

Data will be extracted by two reviewers. Data from the document analysis will be coded using *NVivo*^*TM*^ v14 and framework analysis [35]. Descriptive statistics will be used to summarise the frequencies of content items. All interview transcripts will also be analysed by the same two reviewers, using *NVivo*^*TM*^ v14 and the five-step framework approach [30]. Reviewers will familiarise themselves with the interviews to enable the identification of recurring themes and key ideas. A thematic framework will then be developed jointly by the reviewers – informed by the findings of the Phase 2A survey and document analysis – against which all transcripts will then be systematically indexed and coded [35]. An initial pilot review of five transcripts will then be completed by both reviewers, where each reviewer will independently assign codes to relevant sections of the text. Following the pilot, both reviewers will meet to discuss their coding decisions, resolve discrepancies and update any aspects of the framework where required. After which, the reviewers will meet after every ten transcripts that are coded to assess consistency, until all transcripts have been assessed. Once coded, interview transcripts will be examined for patterns, themes and sub-themes. Interview findings will be integrated with the findings of the document analyses [30].

#### Stream 2B participant eligibility criteria.

For **Stream 2B**, the conveners of IPC courses will be identified from relevant heads of schools and university/institution websites, and other Australian registered training organisations (RTOs) who deliver IPC education and training. At time of writing, there are less than 10 IPC courses available in Australia that meet eligibility requirements. Given the relatively small number, document analysis and key informant interviews for all existing courses will likely be feasible. Monetary incentives will be provided to encourage participation.

#### Stream 2B data collection and sources.

Data pertaining to IPC educational courses will be collected from publicly available university and institutional websites, as well as any physical/digital marketing materials such as pamphlets [30]. Course conveners will be invited to participate in interviews via email, which will include the participant information package with an interview booking link. The interviews will be conducted on Microsoft Teams using a semi-structured interview guide and follow the same process for the interviews in Stream 2A. The interview will primarily comprise open-ended questions to explore participants’ perspectives on current elements of IPC training and programs. Interviews will take 60 minutes to complete and will be recorded and transcribed verbatim. Interview participants will be reimbursed with an AU$50 electronic gift voucher for their participation.

#### Stream 2B data analysis.

Data from the document analysis will be analysed using *NVivo*^*TM*^ v14 and the framework analysis as in Stream 2A [35].

### Phase 3 – e-Delphi

#### Objectives.

1. Establish core requirements for the elements and governance systems of IPC programs in Australian hospitals (**3A**).
2. Establish minimum professional practice and competency standards to underpin ICP practice and education in Australian hospitals (**3B**).

#### Study design.

An e-Delphi will be used to synthesise expert input and determine the core items for standards for both Stream A and B [36]. The e-Delphi will combine the functionality of real-time consensus with iterative survey rounds. This method has been previously employed by members of the research team to identify research priorities and practice standards for emergency nursing in Australia [10,37], and adapting a nursing assessment framework for residential aged care [38].

#### Participants and eligibility criteria.

Panel experts will be convened from a convenience sample of individuals that meet the first criterion, and at least one of the subsequent three criteria, listed below:

- Is a currently employed (or recently employed within the last 12 months) practicing ICP; and
  - Is involved in the practice of IPC in an acute clinical, academic or research setting; or
  - Is responsible for service delivery at a jurisdictional or organisational level; or
  - Is responsible for clinical, education, research and quality management activities related to IPC.

#### Sample size.

A sample size of 338 is required for the e-Delphi process. This was calculated and adjusted using the method to estimate sample size in finite populations shown in Table 2. The calculation assumes a total ACIPC full membership population of 1,468, [39] and an IPC lead population of 1,340, assuming there is at least one IPC lead for each Australian hospital. [31]

**Table 2:** Sample size calculations for the e-Delphi expert panel.

Assumptions	Sample Size Calculation
Z= 1.96, p = 0.5, CI = 0.05 ACIPC Membership=1468 Hospital IPC Lead=1340 Total Population (Pop)=2808	$Sample\;Size=\frac{Z^{2}\times p\times (1-p)}{{CI}^{2}}=\frac{{\left(1.96\right)}^{2}\times 0.5\times (1-0.5)}{{\left(0.05\right)}^{2}}=384$ $Adjusted\;sample\;size=\frac{SS}{1+\frac{SS-1}{Pop}}=\frac{384}{1+\frac{384-1}{2808}}=338$

Z = Z-value, p = % for selecting a choice (50% was chosen as being the most conservative, CI = confidence interval.

### Data collection

Experts will be recruited via email invitation through several participating partner organisations, including the ACIPC, APHA and State and Territory health departments. Email invitations will provide a participant information package, including contact details for the jurisdictional lead investigators, and a link to the e-Delphi. The e-Delphi will be completed using the bespoke Delphi survey tool, *Calibrum Surveylet* (Calibrum International, USA). Each round will be open for eight weeks. Up to three reminder emails will be distributed during this period to encourage participation, with the final email to be sent two days before the close date. The e-Delphi ends once all items have reached consensus.

Questions in Round 1 will be informed by the outcomes of Phases 1 and 2, and piloted by select members of the research team, including expert ICPs, for further refinement [10,37]. The Delphi will have two distinct sections, one for each stream. For Stream A, there will be statements pertaining to participant expectations of IPC program elements and governance, specifying minimal requirements for program contents, structure, evaluation and organisational support. Stream B will include statements about the professional competency of ICPs, focusing on expected clinical skills, education, training and qualifications, as well as the assessment of competence and performance. Experts will rate the importance of each statement on a four-point Likert scale (1 = not important, 4 = very important) [40]. Optional open-ended questions will accompany each statement to allow for additional input. An adjunct demographic survey will capture the expert’s age, experience, professional role and academic qualifications. If the expert is currently working in a hospital, the survey will obtain the general characteristics of their facility, such as size and location.

The e-Delphi will enable experts to revise their responses until the survey closes. During this, panel members will simultaneously see the aggregate of other responses anonymously in real-time, including the distribution of ratings and comments. At the end of each round, a summary of responses will be provided to all participants before starting the subsequent round.

Delphi participants will be reimbursed with an AU$30 electronic gift voucher for each survey round they complete. Participants will be asked to provide their email address when commencing a survey round. Once the survey round has ended, participant email addresses will be separated from survey their responses, and the gift voucher will be sent to the participant via email.

### Data analysis

Sample characteristics and data from each round will be summarised using descriptive statistics. Statistical tests for sample characteristics will be considered to assess the representativeness of the panel. Quantitative data will be imported into *SPSS* v26 to calculate the frequency, median, interquartile range and content validity index for each Likert scale item. Comments from open-ended questions will be analysed by two reviewers from their respective streams using content analysis. Feedback from qualitative data will inform the modification of items that do not reach consensus in the subsequent round.

Consensus will be determined by the level of agreement within the panel using a content validity index (CVI). The CVI for each item will be calculated by the number of participants who gave a positive rating (3 or 4) divided by the total number of participants who responded. Items with a CVI of ≥80% or ≤20% are considered to have reached a high level of consensus and thus accepted by the expert panel. Items that do not reach consensus will be modified based on feedback and forwarded to the next round. Internal consistency will be determined for each round of the e-Delphi survey using the Cronbach alpha coefficient. Differences between item agreement in Delphi rounds will be examined using paired t-tests and chi-squared (χ^2^) tests. A p-value < 0.05 will be considered statistically significant.

### Ethics approval

This research has been approved by the University of Sydney Human Research Ethics Committee (2025/HE000040). In general, this research is a low/negligible risk and involves no high-risk methodologies.

### Ethical considerations

#### Participant recruitment and informed consent.

Participation across all phases of the research is voluntary. Written, verbal or implied consent will be sought from all individuals participating in the surveys and interviews during Phase 2 and Phase 3. For Phase 2 interviews, participant information statements and consent forms will be provided as an attachment to the email invitations. Participants will be given the option to either return a signed electronic copy of the consent form via email or give verbal consent to the interviewer prior to commencing the interview. The interviewer will then complete a physical copy of the consent form, signing as a proxy to acknowledge the verbal consent of the participant. For the survey in Phase 2 and the e-Delphi in Phase 3, participant information statements will also be circulated with the email invitations. Informed consent will be implied through the submission of a completed online survey. Participants will have the right to request access to the information they will have provided to the research team. They will be able to access a copy of the results by direct request to the Chief Investigator.

#### Confidentiality and privacy.

Participant privacy will be protected, with all participants blind copied in email communications related to the project. No personal identifiers will be collected during the data collection. Interview transcripts (Phase 2A and Phase 2B) will be de-identified by investigators. Participants who complete the Phase 2 and/or Phase 3 surveys will be assigned unique IDs. Participants completing each round in the Phase 3 e-Delphi will be required to enter their email address. However, these email addresses will not be linked to the survey responses and will only be used for providing their reimbursement for participation.

All surveys will be completed in either *REDCap*^*TM*^ (Phase 2) or *Calibrum Surveylet* (Phase 3). Once surveys close, data files will be downloaded directly from the survey tool and stored on the Research Data Store (RDS) maintained by the University of Sydney. The RDS is accessible only to study investigators nominated by the Chief Investigator. All results related to participant characteristics will be presented as aggregate data and no data that identifies individual participant(s) will be reported in publications or presentations arising from the study.

#### Safety considerations.

There are no expected risks to participants and participation is voluntary. There will be no direct contact between the research team and participants for the initial invitation in Phases 2 or 3. Invitations to participate will be distributed from the office of the Chief Investigator (Phase 2) or from relevant partner organisations (Phase 3). Subsequent contact is at the discretion of the participant by providing their email address. All data transmission and collection software used are secure, including *REDCap*^*TM*^, *Calibrum Surveylet* and *MS Teams*. No data will be transferred to ACIPC or used in other capacities by any third parties. Only members of the research team will have access to the study data.

#### Data management.

All data will be managed according to the University of Sydney’s Research Data Management Policy and Procedures. All data will be stored on password-protected confidential servers within the University of Sydney, Camperdown Campus in accordance with prevailing legislation policies at both institutions. Access to data files will be provided only to approved members of the research team, authorised by the Chief Investigator. Data will not be used for any additional purposes beyond those described in this study. In accordance with the University of Sydney Policy, records for this study will be stored securely in the RDS for five years following publication of the results before being securely destroyed. 

#### Project timeline.

This research program will commence in January 2025 and will be completed over four years, with activities from Stream A and Stream B completed in parallel. All three phases will be completed within the first three years, with each phase estimated to require approximately one year for completion. The first year will be allocated for the completion of all administrative and recruitment activities. This will be followed by the completion of the Phase 1 integrative literature review, starting in April 2025. The second year will see the completion of Phase 2, with participant recruitment for the surveys and document analysis commencing in January 2026 and expected to take six months to complete, followed by another six months to recruit and complete the key informant interviews. Finally, the Phase 3 e-Delphi will be completed over the third year, which includes time allocated to recruit the expert panel, provide feedback between rounds and develop the survey instruments based on the outcomes of Phase 1 and 2. Participant recruitment for the e-Delphi is estimated to be completed by September 2027 and results will be finalised by the end of December 2027. The fourth and final year, following the completion of Phase 3, will be allocated to the translation and dissemination of the outcomes in the form of formal standards, guides and documentation templates, as well as peer-reviewed publications.

## Discussion

The introduction of national accreditation standards for hospitals in Australia [11,12], as well as professional associations for ICPs [13,14], has been pivotal in the efforts to reduce HAIs, manage AMR, and improving the quality and safety of care of Australian healthcare. However, there remains considerable heterogeneity across jurisdictions in how IPC programs are designed and implemented, both within the broader community, and in hospitals and healthcare service providers [7,15–17]. Existing resources currently lack the information to fully inform the core contents and composition of a comprehensive IPC program, including specifications tailored for different hospital profiles (e.g., size, location, adult or paediatric, specialist services). Similarly, there are few resources that inform the minimum qualifications, training and skills required of the ICP, within either education curricula or formal credentialling requirements.

This critical research will generate a core set of recommendations, in the form of minimum, or ‘core’ requirements for IPC programs and practice in Australian hospitals. Importantly, these recommendations will be evidence-based, consensus-driven by Australia IPC professionals and experts, and be sensitive to key variations in facility characteristics that are most likely to impact practice or implementation. The development of minimum standards will complement and support the existing NSQHS Standards and guidelines for IPC programs, as well as inform the requirements for education, knowledge and competency for ICPs in Australian hospitals. It aims to provide more specified, practical and pragmatic recommendations for the contents and governance of hospital IPC programs, which are currently lacking in available resources. For educators, this study will provide a national framework for the training, education and assessment of ICPs, by informing the core elements of the curriculum for approved IPC courses. For hospitals and healthcare providers, study results will inform the position descriptions for the ICP’s roles and responsibilities and key performance indicators against which they are evaluated. Furthermore, the outcomes of the literature reviews, document analyses and surveys will further inform and contextualise the standards within both national and global efforts, such as the WHO’s minimum requirements for IPC programs [21], the current national standards and guidelines [12,22], the existing credentialling framework and educational offerings [14,23]. This aims to reduce redundancy and ensure the standards are congruent with ongoing initiatives in the broader landscape.

This research capitalises on shared goals with IPC leaders across all Australian States and Territories, national policymakers, professional organisations and health service providers, to urgently deliver new context-specific, evidence-based IPC program and practice standards for all Australian public and private hospitals. Introducing these standards will lead to greater consistency in IPC practice in Australian public and private hospitals. The standards will ensure that IPC staff are well-equipped with the knowledge and skills to implement effective IPC programs themselves and that their organisations are adequately prepared to provide the resources and governance systems.

## Limitations of the design

There are two potential limitations of the research design. Firstly, as Phase 1 involves a literature review to identify current IPC practices and standards for ICPs, there is a possibility that the existing body of literature is small, inconsistent, contradictory, or lacks a sufficient evidence base. Therefore, the baseline for which to compare Australian practices may not provide many additional insights or not reflect Australian practice. Despite this, it is deemed important to collate the evidence and understand these existing practices and standards.

Furthermore, in Phase 3, the sample size calculation estimates that 338 participants will be required for the Delphi process. While this sample size is high, the desire across the sector for developments in these areas and the professional connections maintained by the wide-reaching research team should render this target achievable. Partner organisations involved in the study, including IPC representatives from jurisdictional health departments will assist with recruitment to achieve the target sample size. Response rates will be monitored over the data collection period to regularly evaluate the need to implement additional strategies to improve participation. In the case of persistently low response rates, the target sample size will be reevaluated to prioritize obtaining a representative sample of experts for the Delphi where responses from minimum number of experts from specific populations (e.g., rural hospitals, children’s hospitals) are obtained. This strategy will still allow for the completion of the Delphi with valid results, even if the original target sample size is not met.

## Dissemination plan

The outcomes from each phase of the research will be disseminated in a variety of forums, including open access peer-reviewed scientific journals, conference proceedings, social media, press releases and other local, national and international presentations. All publications will include information on the sources of financial and in-kind support for the research and any potential conflicts of interest. Authorship for publications will be decided prior to each publication and will comply with the International Committee of Medical Journal Editors (ICMJE) guidelines for peer-reviewed publications [41].

## Amendments to the protocol

Major amendments or variations to the research design or methodology, including major delays or termination, will be documented and submitted to the University of Sydney HREC and any other relevant partners or funding agencies for approval. Minor variations that do not materially affect the conditions of the human research ethics approval, cause significant disruptions to the research, or its completion will be documented by the Chief Investigator in an implementation log and submitted as part of an annual progress report to the University of Sydney HREC and other relevant partners or funding agencies.
